# AtMPK1/AtMPK2 Interact with AtVQ25 to Enhance Its Protein Stability and Coordinately Regulate Salicylic Acid-Mediated Leaf Senescence

**DOI:** 10.3390/plants15172726

**Published:** 2026-09-06

**Authors:** Qi Tan, Mengwei Zhang, Yujia Song, Pengcheng Yin, Ke Li, Xiao Meng, Geng Wang, Chunjiang Zhou

**Affiliations:** 1 Ministry of Education Key Laboratory of Molecular and Cellular Biology, Hebei Research Center of the Basic Discipline of Cell Biology, Hebei Collaboration Innovation Center for Cell Signaling and Environmental Adaptation, Hebei Key Laboratory of Molecular and Cellular Biology, College of Life Sciences, Hebei Normal University, Shijiazhuang 050024, China; 18631504498@163.com (Q.T.); zmw2517@163.com (M.Z.); songyujia818@163.com (Y.S.); ypcsjj@163.com (P.Y.); like@hebtu.edu.cn (K.L.); mengxiao2012@163.com (X.M.); 2 Institute of Millet Crops, Hebei Academy of Agriculture and Forestry Sciences, Shijiazhuang 050035, China; 3 College of Life Sciences, Hebei Agricultural University, Baoding 071001, China

**Keywords:** leaf senescence, AtVQ25, AtMPK1/AtMPK2, protein stability, salicylic acid

## Abstract

Leaf senescence is a pivotal developmental program in plants, precisely regulated by diverse signals including salicylic acid (SA). The Arabidopsis VQ protein AtVQ25 has been previously characterized as a positive regulator of SA-mediated leaf senescence, functioning through interaction with AtWRKY53 to relieve the transcriptional self-repression of *AtWRKY53* at its own promoter. However, the upstream regulatory mechanisms governing AtVQ25 itself remain elusive. In this study, a yeast library screening was performed, and the mitogen-activated protein kinases AtMPK1 and AtMPK2 were identified as interacting partners of AtVQ25. The direct physical interaction was validated by yeast two-hybrid (Y2H), luciferase complementation imaging (LCI), pull-down, and co-immunoprecipitation assays (Co-IP). Furthermore, AtVQ25 was shown to be directly phosphorylated by AtMPK1/AtMPK2, which enhanced its protein stability and retarded its degradation, thereby positively modulating leaf senescence progression. Genetic analyses revealed that the function of AtVQ25 in SA-mediated leaf senescence depends on the functional integrity of AtMPK1/AtMPK2. Collectively, these findings establish AtMPK1/AtMPK2 as upstream interactors of AtVQ25 that coordinate SA-mediated leaf senescence through phosphorylation-dependent enhancement of AtVQ25 protein stability, providing novel insights into the upstream regulatory circuitry of VQ proteins.

## 1. Introduction

Leaf senescence demands precise spatiotemporal control, as its timing directly affects nutrient remobilization, reproductive success, and ultimately crop yield. This programmed degeneration, including chlorophyll breakdown, macromolecular hydrolysis, and nutrient export from aging leaves, is an actively regulated process [1,2]. Phytohormones play critical roles in regulating senescence, among which SA exerts a prominent promoting effect by gradually accumulating in senescing leaves and subsequently activating a cascade of senescence-associated genes (*SAGs*) [3]. Genetic evidence indicates that disruption of SA biosynthesis (*sid2* mutant) or signal transduction (*npr1* mutant) results in markedly delayed leaf senescence, and *NahG* transgenic plants expressing SA hydroxylase likewise exhibit senescence-retardation phenotype [3,4]. In recent years, the interactions between SA and ethylene, jasmonic acid, and reactive oxygen species (ROS) signaling have been increasingly uncovered. These pathways cooperate to regulate the onset and progression of senescence [5,6].

Mitogen-activated protein kinase (MAPK) cascades are evolutionarily conserved signaling modules composed of three tiers of phosphorylated kinases—MAPKKKs, MAPKKs, and MAPKs—which transduce extracellular stimuli and developmental cues into intracellular phosphorylation events. The Arabidopsis genome encodes 20 *MAPK* members, which are broadly involved in stress responses, hormone signaling, and developmental regulation [7,8]. MAPK cascades regulate leaf senescence through multiple mechanisms by targeting NPR1, a core component of SA signaling. MPK6 both activates the transcription factor WRKY6 to enhance *NPR1* transcription and modulates thioredoxin Trx h5 to promote NPR1 monomerization and nuclear localization, thereby synergistically enhancing SA-induced leaf senescence. Furthermore, in the MKK4/5-MPK1/2 cascade, MPK1 directly phosphorylates NPR1 and mediates its monomerization, a process that is strictly dependent on SA and NPR1. Additionally, MAPK cascades also modulate leaf senescence via regulation of ROS accumulation [9,10,11,12,13,14,15].

VQ proteins constitute a family of plant-specific transcriptional regulatory cofactors, characterized by a conserved VQ motif (FxxxVQxxTG) that mediates interaction with WRKY transcription factors to modulate their transcriptional activity [16]. Accumulating evidence has demonstrated that VQ proteins are involved in plant defense responses, abiotic stress tolerance, and developmental regulation, with VQ-WRKY interaction emerging as a conserved transcriptional regulatory paradigm in plants [17,18]. Previous studies established that AtVQ25 directly interacts with AtWRKY53, attenuating the binding affinity of AtWRKY53 for W-box elements within its own promoter, thereby relieving *AtWRKY53* transcriptional self-repression and positively regulating SA-mediated leaf senescence [19]. However, the mechanisms governing AtVQ25 protein level during senescence and the possible existence of upstream kinase-mediated stability control have remained unresolved.

In this study, upstream regulators of AtVQ25 were investigated to elucidate the molecular mechanism controlling its protein abundance during senescence. Through yeast library screening, the AtMPK1 and AtMPK2 were identified as candidate interactors of AtVQ25. The direct binding of AtVQ25 with AtMPK1/AtMPK2 was confirmed by a combination of Y2H, LCI, pull-down, and Co-IP. Domain mapping revealed that this interaction primarily relies on the N-terminal and P1 regions of AtVQ25, rather than its conserved VQ domain. Functional studies demonstrated that interaction with AtMPK1/AtMPK2 significantly enhances AtVQ25 protein stability and retards its degradation. In vitro kinase assays further confirmed that AtVQ25 is directly phosphorylated by AtMPK1/AtMPK2, implicating phosphorylation in stability modulation. Genetic analyses revealed that in the *atmpk1atmpk2* mutant background, the premature senescence phenotype induced by *AtVQ25*-overexpressing (OE) lines under SA treatment is substantially suppressed, indicating that AtVQ25 function in SA-mediated leaf senescence depends on the functional integrity of AtMPK1/AtMPK2. Moreover, a potential interaction between AtWRKY53 and AtMPK1/AtMPK2 was uncovered, suggesting the existence of multilayered regulatory networks. Collectively, these findings unveil a novel mechanism by which AtMPK1/AtMPK2 function as upstream stabilizing factors for AtVQ25, offering new perspectives on how MAPK signaling modulates leaf senescence through VQ protein regulation.

## 2. Results

### 2.1. AtVQ25 Directly Interacts with AtMPK1 and AtMPK2

**Figure 1:**
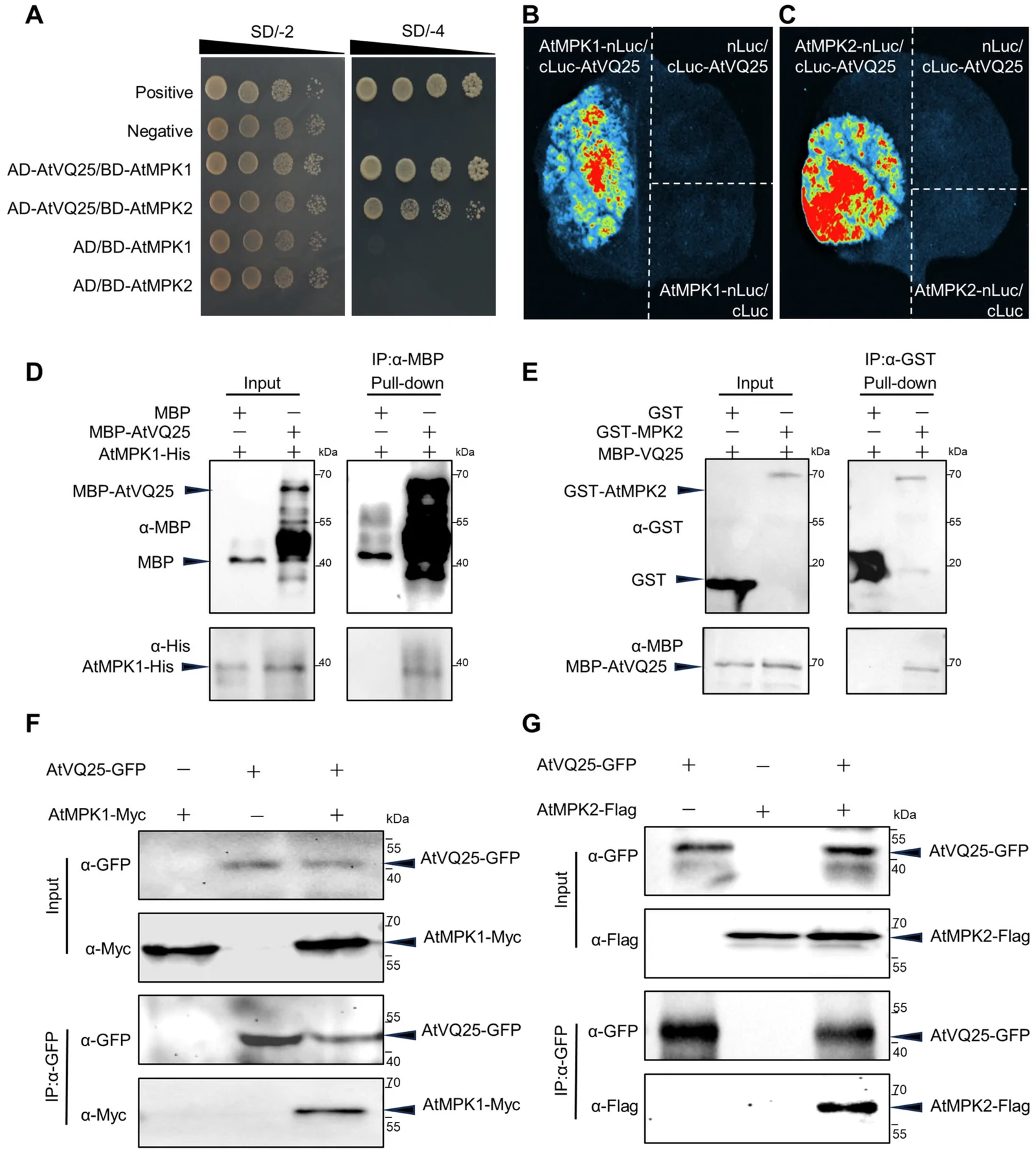
AtVQ25 directly interacted with AtMPK1 and AtMPK2. (**A**) Y2H assay showed the interactions of AtVQ25 with AtMPK1 and AtMPK2. Transformants were grown on SD/–Trp/–Leu or SD/–Trp/–Leu/–His/–Ade. AD-T/BD-p53 and AD-T/BD-Lam served as positive and negative controls, respectively. (**B**,**C**) LCI assay detected the interactions of AtVQ25 with AtMPK1 (**B**) and AtMPK2 (**C**) in *Nicotiana benthamiana* leaves. (**D**,**E**) Pull-down assay demonstrated direct in vitro binding of AtVQ25 to AtMPK1 (**D**) and AtMPK2 (**E**). Proteins were detected by immunoblotting with anti-GST, anti-His, and anti-MBP antibodies as indicated. (**F**,**G**) Co-IP assay validated the in vivo interactions of AtVQ25 with AtMPK1 (**F**) and AtMPK2 (**G**) in *N. benthamiana* leaves co-expressing AtVQ25-GFP with AtMPK1-7Myc6His or AtMPK2-Flag, respectively. Immunoprecipitation was performed using anti-GFP magnetic beads, and immunoblots were probed with anti-GFP, anti-Myc, or anti-Flag antibodies.

To investigate the upstream regulatory mechanism underlying AtVQ25-mediated leaf senescence, yeast library screening was performed to identify potential interacting proteins of AtVQ25, leading to the isolation of AtMPK1 and AtMPK2. The reliability of this screening result was first verified by Y2H assay, in which yeast cells co-transformed with AD-AtVQ25 and BD-AtMPK1 or BD-AtMPK2 grew normally on selective medium (SD/-Trp/-Leu/-His/-Ade), indicating a direct interaction between AtVQ25 and AtMPK1/AtMPK2 in yeast (Figure 1A).

Subsequently, this interaction was validated using LCI assay in *Nicotiana benthamiana* leaves. Detectable luciferase activity was observed upon co-infiltration of AtMPK1-nLuc or AtMPK2-nLuc with cLuc-AtVQ25, whereas no luminescence signal was detected in the negative control groups (Figure 1B,C), confirming the interaction in living plant cells.

To determine whether the interaction reflects direct binding, pull-down assay was performed using recombinant MBP-AtVQ25, AtMPK1-His, and GST-AtMPK2 fusion proteins purified from *E. coli* expression systems. Direct in vitro binding of AtVQ25 to both AtMPK1 and AtMPK2 was demonstrated (Figure 1D,E).

In parallel, the in vivo interaction was examined via Co-IP assay. AtVQ25-GFP was co-expressed with AtMPK1-7Myc6His or AtMPK2-Flag in *Nicotiana benthamiana* leaves, and total protein extracts were subjected to immunoprecipitation using GFP antibody-conjugated magnetic beads. Both AtMPK1-7Myc6His and AtMPK2-Flag were successfully co-precipitated with AtVQ25-GFP (Figure 1F,G), confirming their interaction in the plant cellular context. Furthermore, subcellular localization analysis revealed that AtMPK1 and AtMPK2 are localized to the nucleus (Figure S1A,B), which is consistent with the known nuclear localization of AtVQ25 [19], thus providing a spatial basis for their coordinated regulatory functions within the nucleus. Collectively, these results consistently demonstrate that AtVQ25 directly interacts with AtMPK1 and AtMPK2 both in vitro and in vivo.

### 2.2. Differential Domain Requirements for AtVQ25 Interaction with AtMPK1 and AtMPK2

**Figure 2:**
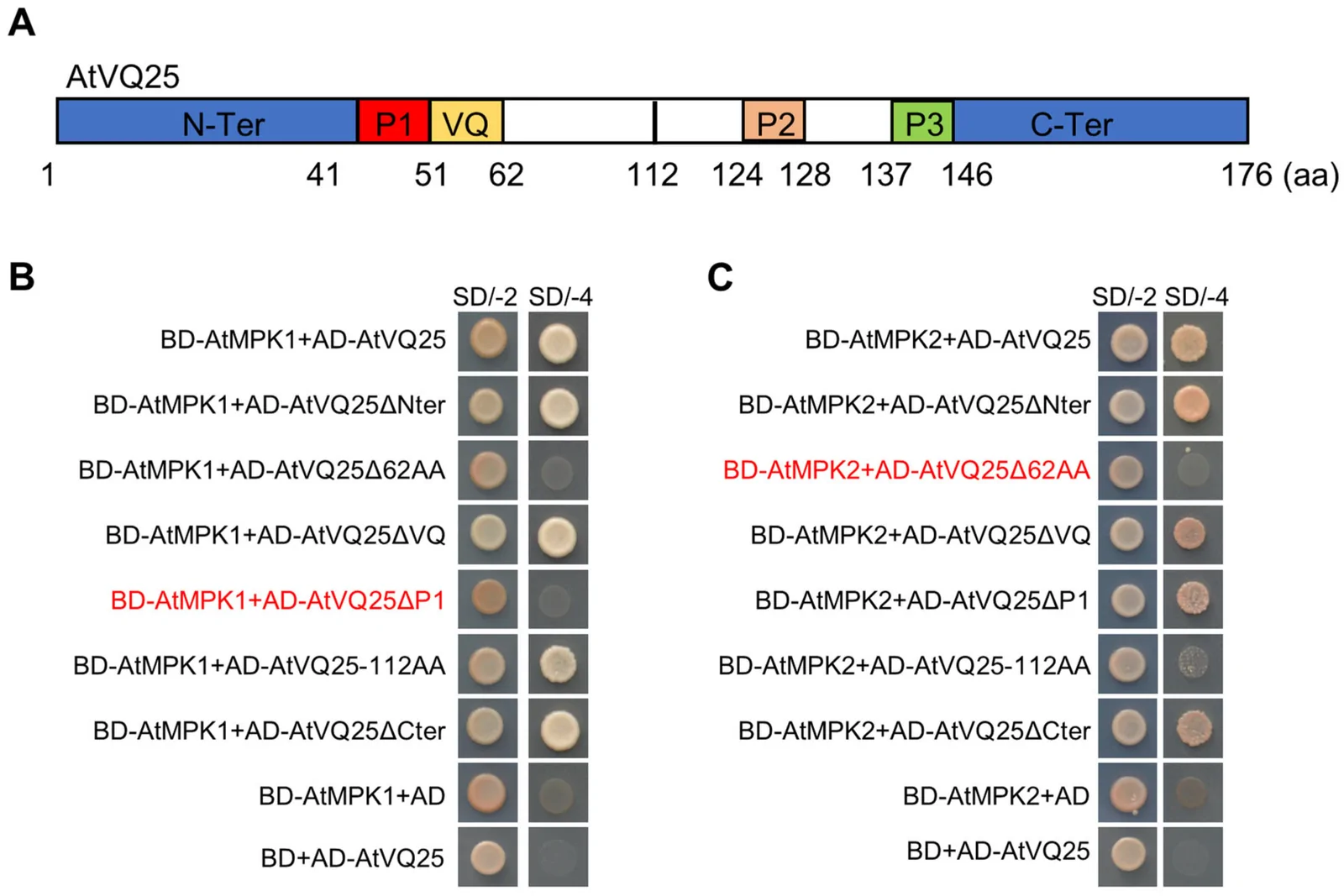
Domain mapping of the AtVQ25–AtMPK1/AtMPK2 interactions. (**A**) Schematic representation of AtVQ25 protein domains. Bioinformatics analysis revealed that AtVQ25 contains an N-terminal region (amino acids 1–41), P1 domain (aa 41–51), P2 domain (aa 124–128), P3 domain (aa 137–146), a conserved VQ motif (aa 51–62), and a C-terminal region (aa 146–176). Δ62 denoted a truncated mutant lacking the first 62 amino acids. (**B**) The interaction between AtMPK1 and AtVQ25 depended on the P1 domain. Y2H assay was performed with various truncated forms of AtVQ25 against AtMPK1 on SD/–Trp/–Leu and SD/–Trp/–Leu/–His/–Ade media. (**C**) The interaction between AtMPK2 and AtVQ25 required the N-terminal 62 amino acids. Y2H assay was performed with various truncated forms of AtVQ25 against AtMPK2 on the same media. All experiments were repeated at least three times with similar results.

To delineate the key domains of AtVQ25 that mediate its interaction with AtMPK1 and AtMPK2, bioinformatics analysis of the AtVQ25 amino acid sequence was performed using the NCBI protein database (https://www.ncbi.nlm.nih.gov/protein/, accessed on 5 March 2023), which predicted an N-terminal region, three potential protein interaction-dependent sites designated P1, P2, and P3, and a conserved VQ domain (Figure 2A). These predictions were subsequently tested via Y2H assay using a series of deletion mutants.

The interaction with AtMPK1 was completely abolished by deletion of the N-terminal 62 amino acids of AtVQ25, which encompass the N-terminus, the P1 domain, and the VQ domain. Further dissection revealed that the binding between AtVQ25 and AtMPK1 was substantially weakened by deletion of the P1 domain alone, indicating that the P1 domain is critical for maintaining this interaction (Figure 2B). Notably, the AtVQ25-AtMPK1 interaction was not affected by deletion of the VQ domain alone, demonstrating that this interaction is independent of the conserved VQ domain of AtVQ25.

In contrast, the interaction between AtVQ25 and AtMPK2 was insensitive to deletion of any single domain. The interaction was substantially diminished only by simultaneous deletion of the N-terminal 62 amino acids (Figure 2C). Similarly, the AtVQ25-AtMPK2 interaction was independent of the integrity of the VQ domain. These results indicate that the AtVQ25-AtMPK2 interaction involves cooperative contributions from multiple domains and exhibits a distinct interaction mode from that of AtVQ25-AtMPK1.

### 2.3. AtMPK1/AtMPK2 Enhance AtVQ25 Protein Stability Through Phosphorylation

**Figure 3:**
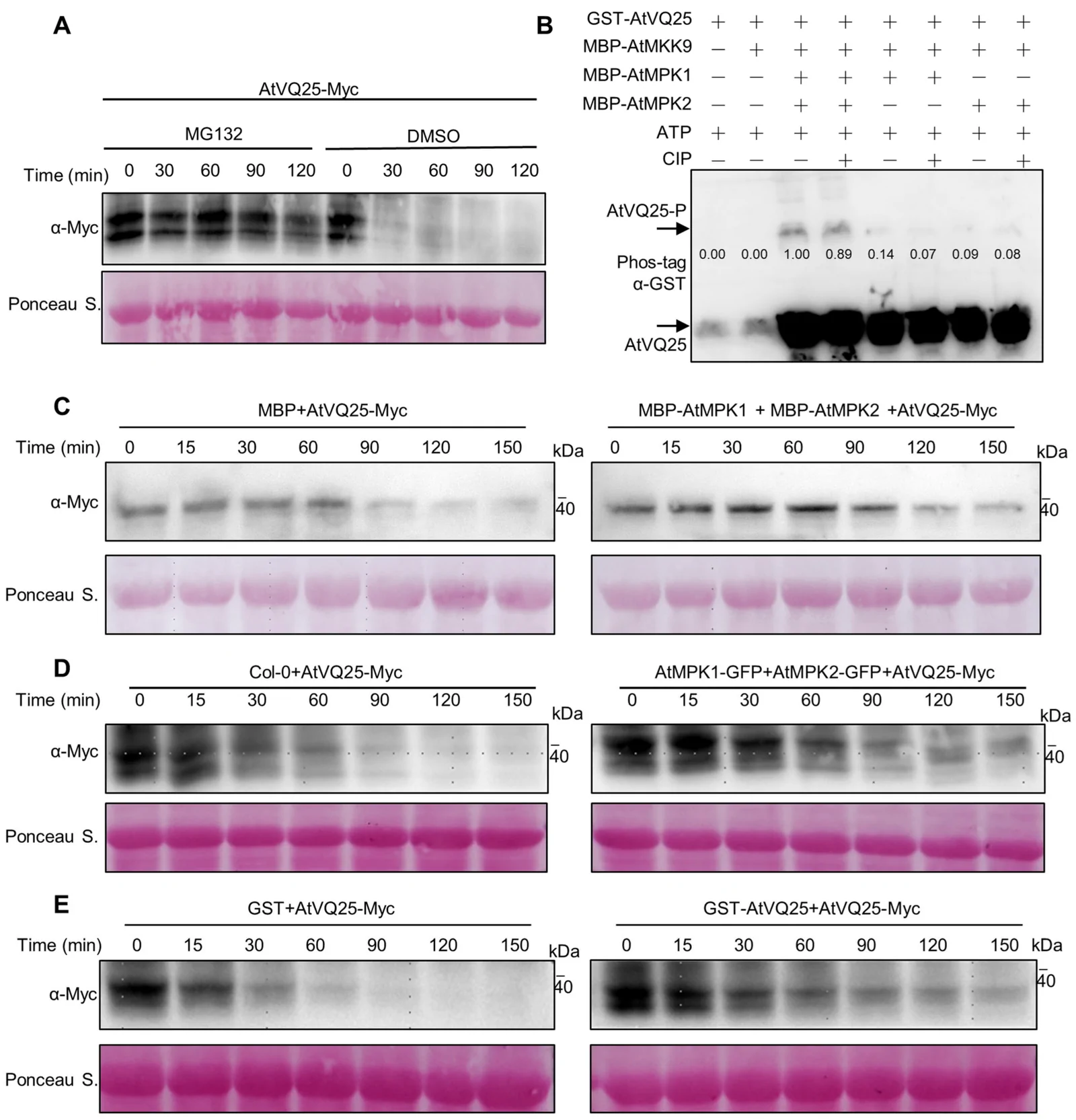
AtMPK1 and AtMPK2 enhance AtVQ25 protein stability via phosphorylation. (**A**) MG132 inhibits AtVQ25 degradation. Total protein extracts from AtVQ25-7Myc6His overexpression plants were incubated with MG132 or an equal volume of DMSO (control) for 0, 30, 60, 90, and 120 min. AtVQ25-7Myc6His protein levels were detected with anti-Myc antibodies. Ponceau S. staining served as a loading control. (**B**) In vitro phosphorylation assays using Phos-tag showed that AtMPK1 and AtMPK2 phosphorylate AtVQ25. Purified GST-AtVQ25 was incubated with MBP-AtMPK1 or MBP-AtMPK2 in the presence of ATP, with AtMKK9 included as an upstream activating kinase. The addition of CIP reduced the phosphorylation-induced mobility shifts. The numbers below each lane represent relative band intensities (gray values), indicating the phosphorylation levels. A no-kinase control was used as a negative control. Reaction products were resolved by Phos-tag SDS-PAGE and immunoblotted with anti-GST antibodies; arrowheads indicate phosphorylation-induced mobility shifts. (**C**,**D**) AtMPK1/AtMPK2 suppressed AtVQ25 degradation. Total proteins from AtVQ25-7Myc6His OE plants were mixed with purified MBP-AtMPK1, MBP-AtMPK2, or MBP control (**C**), or with total proteins from Col-0, AtMPK1-GFP OE, or AtMPK2-GFP OE plants (**D**), and incubated at 25 °C for 0, 15, 30, 60, 90, 120, and 150 min. Residual AtVQ25-7Myc6His was detected by anti-Myc immunoblotting, with Ponceau S. staining as loading control. (**E**) Exogenous GST-AtVQ25 protein inhibited AtVQ25-7Myc6His degradation. GST-AtVQ25 or GST control was added to total protein extracts from AtVQ25-7Myc6His OE plants and incubated at 25 °C for 150 min. Remaining AtVQ25-7Myc6His levels were assessed by anti-Myc immunoblotting; Ponceau S. staining confirmed equal loading. All experiments were repeated at least three times with similar results.

To investigate the potential degradation pathway of AtVQ25, total protein extracts from AtVQ25-7Myc6His overexpressing plants were treated with the proteasome-specific inhibitor MG132. MG132 treatment significantly retarded the degradation of AtVQ25-7Myc6His, whereas the protein level in the DMSO control group gradually decreased over time (Figure 3A), indicating that AtVQ25 degradation is at least partially mediated by the 26S proteasome pathway.

Given that MAPKs typically regulate substrate function through phosphorylation, the direct phosphorylation of AtVQ25 by AtMPK1 and AtMPK2 was investigated. As MAPK activation generally depends on upstream phosphorylation by MAPKKs, the STRING protein interaction database (https://string-db.org/, accessed on 21 February 2024) was first employed to predict candidate upstream activating kinases for AtMPK1 and AtMPK2, and AtMKK9 was thereby identified as a predicted interactor for both kinases (Figure S2A,B). Accordingly, AtMKK9 was included in the in vitro kinase reaction system to activate AtMPK1 and AtMPK2. Purified GST-AtVQ25 was incubated with MBP-AtMPK1 or MBP-AtMPK2 in reaction buffer containing ATP, and the resulting phosphorylation was detected via Phos-tag assay. Compared with the kinase-free control, the addition of either AtMPK1 or AtMPK2 resulted in prominent mobility-shifted bands of AtVQ25 (Figure 3B). To confirm the specificity of these shifted bands, CIP (calf intestinal alkaline phosphatase) was added to the reaction mixtures as a phosphatase inhibitor, which markedly reduced the intensities of the shifted bands, confirming that the mobility shifts were phosphorylation-dependent. The relative band intensities (gray values) shown below each lane reflect the phosphorylation levels. These results demonstrated that AtVQ25 was directly phosphorylated by both kinases.

The effect of AtMPK1 and AtMPK2 on AtVQ25 protein stability was next verified. Total protein extracts from AtVQ25-7Myc6His-overexpressing plants were co-incubated with purified MBP-AtMPK1, MBP-AtMPK2, or MBP control protein. Both MBP-AtMPK1 and MBP-AtMPK2 significantly suppressed the degradation of AtVQ25-7Myc6His, whereas the MBP control exerted no such effect (Figure 3C). To further validate whether this effect is mediated by AtMPK1/AtMPK2 in plant cells, total protein extracts from AtVQ25-7Myc6His-overexpressing plants were mixed with total protein extracts from Col-0, AtMPK1-GFP and AtMPK2-GFP OE plants, and the protein levels of AtVQ25-7Myc6His were determined. The results demonstrated that protein extracts from AtMPK1-GFP and AtMPK2-GFP OE plants significantly retarded the degradation of AtVQ25-7Myc6His compared with Col-0 extracts (Figure 3D). Additionally, increasing the amount of exogenous GST-AtVQ25 in the reaction system competitively delayed AtVQ25-7Myc6His degradation (Figure 3E).

Collectively, these results demonstrate that AtMPK1 and AtMPK2 directly interact with and phosphorylate AtVQ25, thereby enhancing its protein stability and retarding its degradation.

### 2.4. AtMPK1 and AtMPK2 Participate in Leaf Senescence Regulation

**Figure 4:**
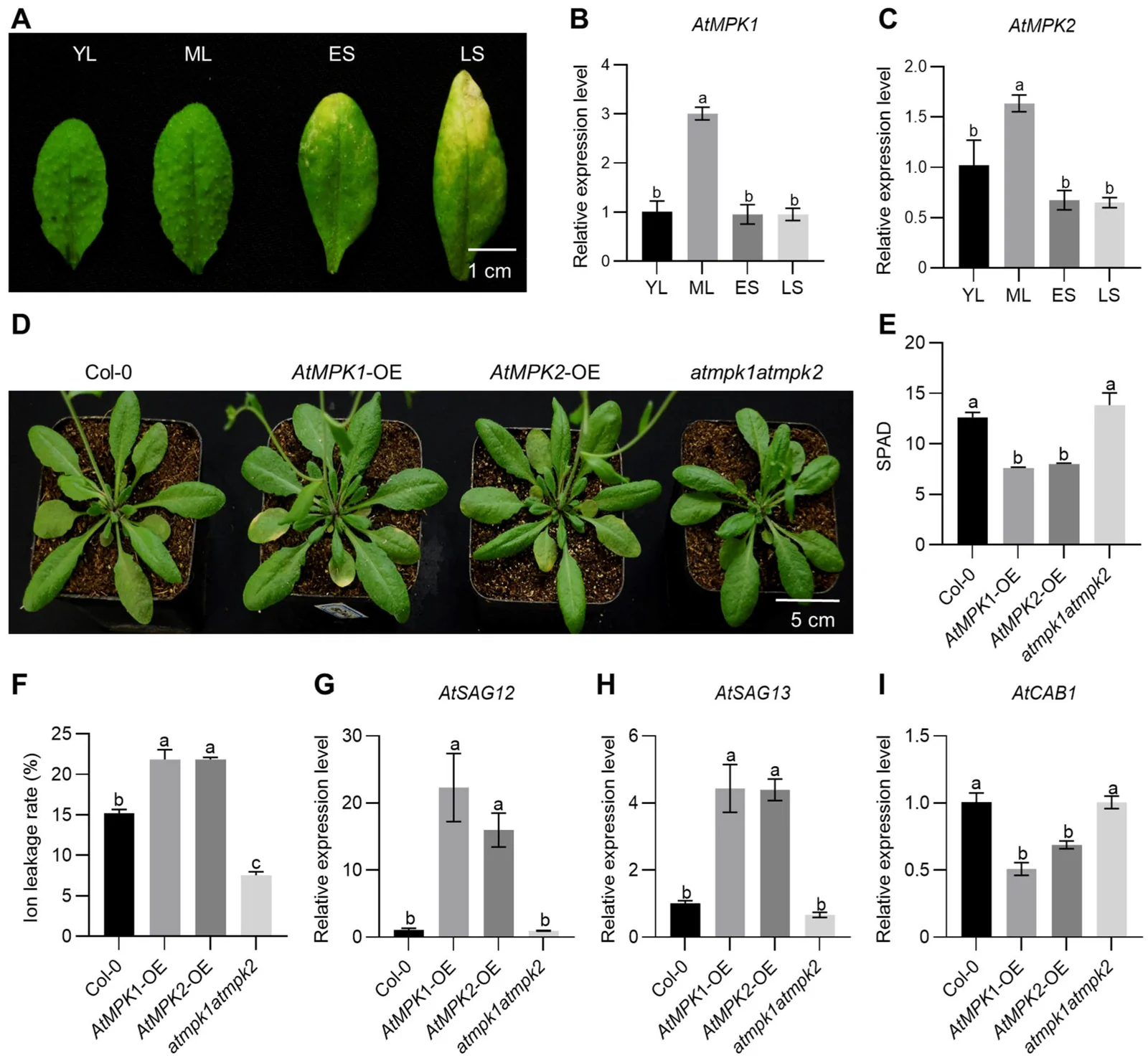
AtMPK1 and AtMPK2 positively regulate leaf senescence. (**A**–**C**) Expression patterns of *AtMPK1* and *AtMPK2* at different developmental stages in Arabidopsis. Total RNA was extracted from young leaves (YL), mature leaves (ML), early senescence leaves (ES) and late senescence leaves (LS), Scale bar = 1 cm (**A**), and relative transcript levels of *AtMPK1* (**B**) and *AtMPK2* (**C**) were determined by qRT-PCR. (**D**) The 6th and 7th rosette leaves phenotypes of 4-week-old Col-0, *atmpk1atmpk2*, *AtMPK1*-OE, and *AtMPK2*-OE plants. Scale bar = 5 cm. (**E**,**F**) SPAD (**E**) and ion leakage rate (**F**) of the 6th and 7th rosette leaves from the plants shown in (**D**). (**G**–**I**) Relative expression levels of three senescence-associated genes, *AtSAG12* (**G**), *AtSAG13* (**H**), and *AtCAB1* (**I**), in the 6th and 7th rosette leaves of the same plants. All experiments were repeated at least three times with similar results. Different letters above bars denote significant differences (*p* < 0.05) as determined by one-way ANOVA followed by Tukey’s HSD post-hoc test.

To explore the biological functions of AtMPK1 and AtMPK2 in leaf senescence, their expression patterns across different developmental stages in Arabidopsis leaves were analyzed. Quantitative real-time PCR (qRT-PCR) analysis revealed that transcript levels of both *AtMPK1* and *AtMPK2* peaked at the mature leaf stage and subsequently declined with advancing senescence (Figure 4A–C), a temporal expression pattern highly similar to that of *AtVQ25* [19].

Phenotypic observation revealed that the *atmpk1atmpk2* exhibited a delayed leaf senescence phenotype, with significantly higher chlorophyll content and lower ion leakage rates in 6th and 7th rosette leaves compared with Col-0. Conversely, *AtMPK1*-OE or *AtMPK2*-OE displayed mild premature senescence phenotypes, characterized by reduced chlorophyll content and elevated ion leakage rates in 6th and 7th rosette leaves. Expression levels of senescence marker genes *AtSAG12*, *AtSAG13*, and *AtCAB1* were consistent with the observed phenotypes (Figure 4D–I). Collectively, these results demonstrate that *AtMPK1* and *AtMPK2* positively regulate leaf senescence progression.

### 2.5. AtVQ25 Cooperates with AtMPK1/AtMPK2 to Regulate SA-Mediated Leaf Senescence

Previous study demonstrated that the *atvq25* loss-of-function mutant exhibits delayed leaf senescence and is insensitive to SA-induced leaf senescence, thereby establishing *AtVQ25* as a positive and necessary regulator of SA-mediated leaf senescence [19]. The upstream regulatory mechanism governing AtVQ25 protein stability during this process was further investigated in the present study.

**Figure 5:**
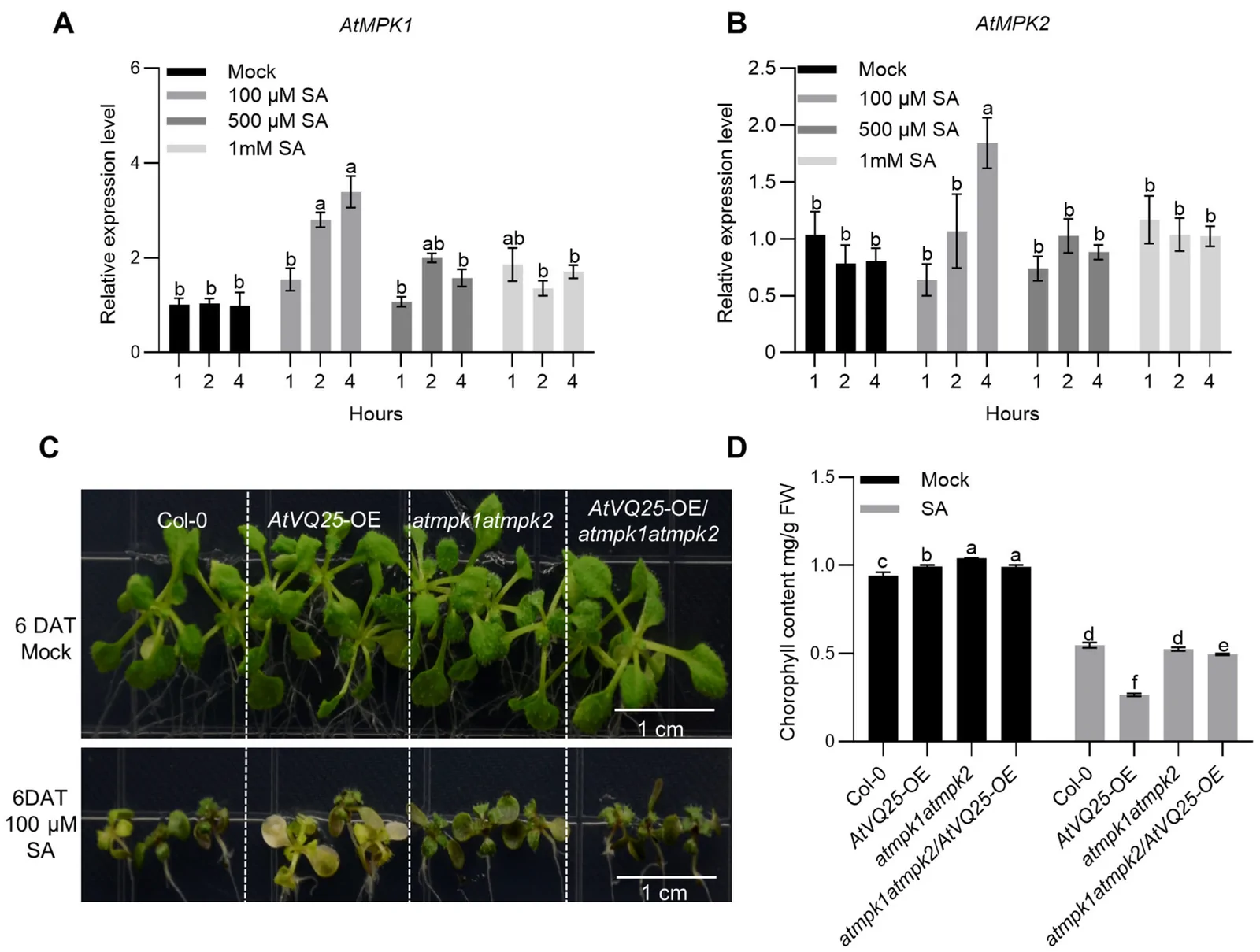
AtVQ25 cooperated with AtMPK1/AtMPK2 to modulate SA-mediated leaf senescence. (**A**,**B**) Responses of *AtMPK1* (**A**) and *AtMPK2* (**B**) to SA treatment. Detached leaves from 3-week-old wild-type plants were treated with 0, 100, 500 μM, or 1 mM SA for 4 h, and relative transcript levels were measured by qRT-PCR. (**C**) Phenotypes of Col-0, *atmpk1atmpk2*, *AtVQ25*-OE, and *AtVQ25*-OE/*atmpk1atmpk2* seedlings grown on MS medium with or without 100 μM SA for 6 d. Scale bar = 1 cm. (**D**) Chlorophyll content of the corresponding genotypes under SA treatment shown in (**C**). All experiments were repeated at least three times with similar results. Different letters above bars denote significant differences (*p* < 0.05) as determined by one-way ANOVA followed by Tukey’s HSD post-hoc test.

Previous studies have demonstrated the involvement of AtMPK1 and AtMPK2 in SA-mediated leaf senescence regulation [12]. The response of *AtMPK1* and *AtMPK2* to SA treatment was examined. Detached leaves from 3-week-old wild-type plants were treated with varying concentrations of SA for 4 h. Expression of both *AtMPK1* and *AtMPK2* was significantly induced by 100 μM SA, whereas the inductive effect was attenuated at 500 μM and 1 mM SA (Figure 5A,B).

To examine the individual contributions of *AtMPK1* and *AtMPK2* to SA sensitivity, the phenotypes of the single mutants *atmpk1* and *atmpk2* under SA treatment were analyzed. Under 100 μM SA, both single mutants, as well as the *atmpk1atmpk2* double mutant, displayed SA sensitivity comparable to that of Col-0 (Figure S3).

To investigate the genetic relationship between *AtVQ25* and *AtMPK1*/*AtMPK2* in SA response, seedlings of Col-0, *atmpk1atmpk2*, *AtVQ25*-OE, and *AtVQ25*-OE/*atmpk1atmpk2* were grown on MS medium supplemented with 100 μM SA for 6 days. All genotypes exhibited growth inhibition and chlorosis upon SA treatment. However, *AtVQ25*-OE displayed the highest SA sensitivity with the most severe chlorosis, whereas *atmpk1atmpk2* exhibited marked insensitivity. Importantly, the phenotype of *AtVQ25*-OE/*atmpk1atmpk2* under SA treatment more closely resembled that of *atmpk1atmpk2* than that of *AtVQ25*-OE (Figure 5C), and this observation was corroborated by chlorophyll content measurements (Figure 5D). These results indicate that *AtVQ25* function in SA response depends on the functional integrity of *AtMPK1* and *AtMPK2*, and that these components coordinately participate in SA-mediated leaf senescence regulation.

### 2.6. AtWRKY53 Exhibits Potential Interaction with AtMPK1/AtMPK2

Given that AtVQ25 cooperates with AtWRKY53 in regulating SA-mediated leaf senescence [19], and that AtVQ25 interacts with and is stabilized by AtMPK1 and AtMPK2, the potential relationship between AtWRKY53 and AtMPK1/AtMPK2 was further investigated. In Y2H assay, growth on selective medium was observed for yeast cells co-transformed with AD-AtWRKY53 and BD-AtMPK1 or BD-AtMPK2, indicating a potential interaction between AtWRKY53 and AtMPK1/AtMPK2 (Figure S4A). Multiple potential phosphorylation sites were predicted in the full-length AtWRKY53 and AtVQ25 proteins using the PhosPhAt 4.0 database (https://phosphat.uni-hohenheim.de, accessed on 12 June 2024) and NetPhos-3.1 server (https://services.healthtech.dtu.dk/services/NetPhos-3.1/, accessed on 12 June 2024). Sites predicted by both tools were considered high-confidence sites. For AtVQ25, the high-confidence predicted phosphosites included serine residues S12, S121, S122, S155, and S156 (Figure S4B–D). Nevertheless, these predictions necessitate further validation via in vivo and in vitro phosphorylation assays.

## 3. Discussion

### 3.1. Discovery of AtMPK1/AtMPK2 as Upstream Interactors of AtVQ25 and Its Implications

A direct protein interaction between AtVQ25 and AtMPK1/AtMPK2 was identified through yeast library screening and validated by multiple independent assays, revealing an upstream kinase layer that directly targets a VQ family protein. This finding expands the known regulatory framework of VQ proteins, which have been primarily characterized as transcriptional cofactors interacting with WRKY transcription factors via the conserved VQ domain [17,18,20]. Notably, the interaction with AtMPK1/AtMPK2 was found to be independent of the VQ domain, instead relying on distinct regions within AtVQ25, indicating that AtVQ25 serves as a multidomain signaling hub that separates upstream kinase docking from downstream transcription factor recruitment.

The observation that AtMPK1/AtMPK2 phosphorylate AtVQ25 and enhance its protein stability provides a mechanistic link between MAPK signaling and the VQ-WRKY transcriptional module. Given that AtVQ25 has been shown to relieve the transcriptional self-repression of *AtWRKY53* during SA-mediated leaf senescence [19], the phosphorylation-dependent stabilization of AtVQ25 by upstream MAPKs may represent a critical control point for fine-tuning the duration and amplitude of senescence-associated transcriptional outputs. This arrangement establishes the MAPK–VQ–WRKY cascade as a phosphorylation-responsive mechanism, where environmental or developmental signals perceived by MAPK cascades can be relayed to downstream WRKY targets through changes in the abundance of the VQ cofactor. Furthermore, these findings raise the possibility that other VQ family members may employ non-canonical interaction modes beyond the VQ domain to receive upstream signals, implying previously unappreciated functional diversification among VQ proteins. Notably, our detection of the AtVQ25–AtMPK1/AtMPK2 interaction contrasts with a previous yeast two-hybrid screening that failed to detect it [21]. This discrepancy likely reflects differences in vector systems, yeast strains, and screening stringency, which may mask weak interactions. Importantly, we validated the interaction by four independent assays, including in vivo LCI and Co-IP, supporting its biological relevance.

### 3.2. Possible Mechanisms by Which AtMPK1/AtMPK2 Enhance AtVQ25 Protein Stability

Based on the observed phosphorylation and stabilization of AtVQ25 by AtMPK1/AtMPK2, together with its proteasome-dependent degradation, the possible molecular mechanisms underlying this regulation are discussed below.

One possibility is that phosphorylation directly alters surface charge distribution or induces conformational changes that mask internal degradation signals, thereby interfering with E3 ubiquitin ligase recognition and ubiquitination efficiency [14,22]. The presence of multiple predicted phosphosites within the N-terminal/P1 interaction region is consistent with this model, although the specific sites and their functional contributions require further validation.

Another possibility is that MPK binding itself, independent of phosphorylation, could allosterically remodel AtVQ25 conformation and physically shield degradation motifs from proteasomal recognition [23]. Whether this effect is accompanied by altered ubiquitination levels remains to be determined.

A third possibility is that these mechanisms act synergistically: phosphorylation may enhance the binding affinity between MPKs and AtVQ25, thereby amplifying the conformational masking effect. The differential domain requirements observed for AtMPK1 versus AtMPK2 further suggest that the relative contribution of each mechanism may vary among different MAPK members, possibly reflecting distinct substrate phosphosite preferences [24,25]. Consistent with this view, the weaker AtVQ25 signals observed in the kinase-free control lanes likely reflect enhanced degradation of the unphosphorylated protein (Figure 3B), further supporting that MPK-mediated phosphorylation protects AtVQ25 from proteasomal breakdown.

Functionally, MPK-mediated stabilization of AtVQ25 is predicted to sustain or increase AtVQ25 protein levels upon SA signaling, thereby reinforcing its cooperative action with AtWRKY53 and promoting senescence-associated gene expression. Future identification of the responsible E3 ligase, assessment of phosphorylation effects on ubiquitination, and generation of phosphosite mutants will be essential to fully elucidate the underlying molecular mechanism.

### 3.3. Differential SA Sensitivity of AtVQ25 and AtMPK1/AtMPK2 and the Regulatory Model

Distinct SA concentration sensitivities were observed between AtMPK1/AtMPK2 and AtVQ25 (Figure 5A,B) [19], with the former induced at low SA levels and the latter requiring higher concentrations for transcriptional activation. This differential responsiveness suggests a two-tiered regulatory logic: low SA levels first activate upstream MAPKs, which phosphorylate and stabilize AtVQ25, thereby initiating senescence-associated transcriptional outputs; at elevated SA concentrations, AtVQ25 transcription is additionally induced, further amplifying the signal. Such a “phosphorylation first, transcription second” mode parallels established MAPK-substrate regulatory paradigms, such as the MPK3/6-mediated regulation of ethylene response factors [15,26].

From a developmental standpoint, the gradual accumulation of endogenous SA with leaf age [27] may coordinately engage AtMPK1/AtMPK2 through dual routes: transcriptional upregulation and post-translational activation via upstream MAPKKs such as AtMKK9. Consistent with this, STRING-based prediction and in vitro phosphorylation assays placed AtMKK9 upstream of AtMPK1/AtMPK2 in the cascade leading to AtVQ25 phosphorylation. Given that AtMKK9 has been implicated in age-dependent and dark-induced senescence, based on this, age-related or SA signal activated AtMKK9, which in turn phosphorylated AtMPK1/AtMPK2, stabilized AtVQ25, and ultimately modulates AtWRKY53-mediated *AtSAGs* expression. Genetic evidence further supports this hierarchy, as the SA-hypersensitive phenotype of *AtVQ25*-OE was fully suppressed in the *atmpk1atmpk2* background, indicating that the function of AtVQ25 in SA response is strictly dependent on AtMPK1/AtMPK2 integrity. Future genetic dissection of the AtMKK9–AtMPK1/AtMPK2 relationship in senescence will be required to consolidate the proposed cascade.

### 3.4. Biological Significance of Differential Domain Requirements Between AtMPK1 and AtMPK2

Although AtMPK1 and AtMPK2 share high sequence homology and overlapping expression patterns, their binding to AtVQ25 relies on markedly distinct region requirements—the P1 domain alone is critical for AtMPK1, whereas AtMPK2 requires cooperative contributions from both the N-terminal and P1 regions. This divergence is unlikely to arise from differences in spatiotemporal distribution, but rather may reflect intrinsic differences in substrate recognition mechanisms between the two MAPKs.

A plausible explanation is that subtle variations in the kinase domain or substrate-binding groove, despite overall high similarity, create distinct contact interfaces for AtVQ25. AtMPK1 may impose a stricter conformational requirement for the P1 domain, while AtMPK2 might depend on combined interactions across multiple sites to achieve stable binding. Together with the VQ-domain-independent nature described above, the differentiated binding modes of the two MAPKs highlight how homologous kinases achieve functional specificity toward a shared substrate through discrete recognition of substrate regions. However, these hypotheses still await further experimental proof.

### 3.5. Potential Significance of AtWRKY53-AtMPK Interaction and the Multilayered Regulatory Model

**Figure 6:**
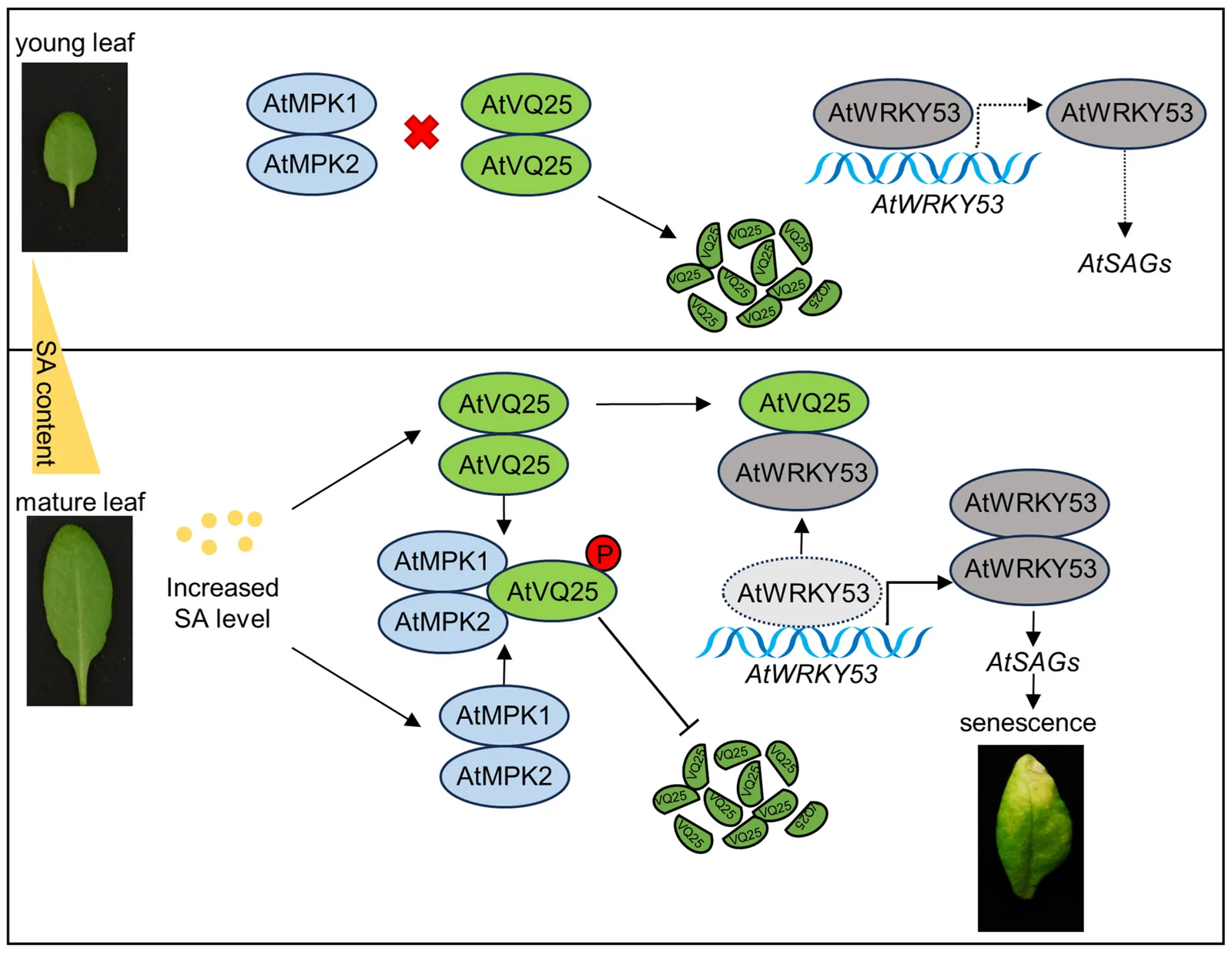
Model of the AtMPK1/AtMPK2–AtVQ25 module in SA-mediated leaf senescence. In young leaves, endogenous SA levels are low, and both the expression and activity of AtMPK1/AtMPK2 are maintained at basal levels. AtVQ25 protein is maintained at low levels and is continuously degraded. Transcription of *AtWRKY53* is repressed by autoinhibition of its own promoter, thereby preventing activation of downstream *AtSAGs*, and normal leaf growth is sustained. In senescent leaves, with increasing leaf age, endogenous SA gradually accumulates, and the expression of *AtMPK1/AtMPK2* is activated by this accumulation. AtVQ25 is directly phosphorylated by activated AtMPK1/AtMPK2, enhancing its protein stability and leading to its accumulation. The self-repression of the *AtWRKY53* promoter is relieved by interaction with stabilized AtVQ25, thereby promoting transcriptional activation of downstream *AtSAGs*, which ultimately results in the initiation and progression of the leaf senescence program.

The potential interaction between AtWRKY53 and AtMPK1/AtMPK2 (Figure S4A), together with the presence of predicted phosphosites in AtWRKY53, raises the possibility that AtWRKY53 may also serve as a direct target of MAPK phosphorylation, consistent with previous reports on MAPK-mediated regulation of WRKY transcription factors in Arabidopsis [9,28]. Integrating these findings with the established AtVQ25-AtWRKY53 working model [19], an updated regulatory framework is proposed (Figure 6).

In young leaves, where endogenous SA levels are low and AtMPK1/AtMPK2 activity remains basal, AtVQ25 protein is maintained at low abundance due to constitutive proteasomal degradation. Under these conditions, AtWRKY53 is subjected to transcriptional self-repression, and senescence-associated genes remain inactive, thereby preserving normal leaf growth.

With advancing leaf age, endogenous SA gradually accumulates, leading to transcriptional upregulation of AtMPK1/AtMPK2 and potentially enhanced kinase activity through upstream MAPKKs such as AtMKK9. Activated AtMPK1/AtMPK2 then phosphorylate and stabilize AtVQ25, enabling its accumulation. Stabilized AtVQ25 subsequently interacts with AtWRKY53 to relieve its promoter self-repression, thereby activating senescence-associated gene expression and promoting leaf senescence progression.

In addition, the observed yeast two-hybrid interaction between AtWRKY53 and AtMPK1/AtMPK2 suggests the possibility of direct MPK action on AtWRKY53, a hypothesis that awaits experimental verification. If confirmed, this would establish a multilayered MAPK–VQ–WRKY regulatory network, in which AtMPK1/AtMPK2 serve as signaling nodes linking developmental and SA cues to the VQ-WRKY transcriptional module, while AtVQ25 functions as a central hub—receiving upstream kinase signals through its N-terminal/P1 regions and modulating downstream transcription factors via its VQ domain—thereby enabling sensing, amplification, and execution of senescence signals.

## 4. Materials and Methods

### 4.1. Plant Materials and Growth Conditions

The *Arabidopsis thaliana* ecotype Columbia-0 (Col-0) was used as the wild-type background for all experimental materials. *Nicotiana benthamiana* plants were used for transient expression assays. The T-DNA insertion mutants *atmpk1* (*AT1G10210*; SALK_048910) and *atmpk2* (*AT1G59580*; SALK_047422) were gifted from Prof. Chunguang Zhang. Seeds of *AtVQ25*-OE lines have been described previously [19]. The primers used are listed in Table S1.

For seed surface sterilization, all Arabidopsis seeds were sterilized in a laminar flow hood with 75% (*v*/*v*) ethanol twice for 2 min each, followed by 95% (*v*/*v*) ethanol for 2 min. The sterilized seeds were sown on 1/2 Murashige and Skoog (MS) medium (0.8% agar, 1% sucrose, pH 5.8) and stratified at 4 °C in the dark for 2 to 3 days to synchronize germination. Subsequently, the seeds were transferred to a growth chamber set at 22 °C with 60% relative humidity and a 16-h light/8-h dark photoperiod. Seven-day-old seedlings were transplanted into nutrient soil for further growth.

### 4.2. Vector Construction and Genetic Transformation in Arabidopsis

The coding sequences (CDSs) of *AtMPK1* and *AtMPK2* were amplified from Arabidopsis cDNA and cloned into the T-Vector pMD™19 (TAKARA, 3271, Kusatsu, Shiga, Japan), which were further ligated into different vectors for subsequent experiments. Primers used for gene cloning and vector construction are listed in Table S1. Agrobacterium tumefaciens-mediated transformation was performed using the floral dip method with strain GV3101. Transgenic Arabidopsis plants were screened on medium containing hygromycin.

### 4.3. RNA Extraction and Transcript Analysis

Rosette leaves of *Arabidopsis thaliana* were collected at four distinct developmental stages: young leaves (YL), which were approximately half the size of fully expanded leaves; mature leaves (ML), which were fully expanded with no visible yellowing; early senescence leaves (ES), which exhibited less than 25% leaf area yellowing; and late senescence leaves (LS), which showed more than 50% leaf area yellowing. The 6th and 7th rosette leaves were harvested for each stage. All samples were immediately frozen in liquid nitrogen and stored at −80 °C until RNA extraction.

Total RNA was extracted from *Arabidopsis* leaves and seedlings using Trizol reagent (TAKARA, 9109) and reverse-transcribed into cDNA using HiScript II QRT SuperMix (Vazyme, R222-01, Nanjing, Jiangsu, China) following the manufacturer’s instructions. qRT-PCR was performed on a Bio-Rad CFX96 system using ChamQ Universal SYBR qPCR Master Mix (Vazyme, Q711-02). Each 10-μL reaction contained 5 μL of 2× SYBR qPCR Master Mix, 0.2 μL of each primer (10 μM), 1 μL of diluted cDNA, and 3.6 μL of nuclease-free water. The thermal cycling conditions were as follows: initial denaturation at 95 °C for 30 s, followed by 35 cycles of 95 °C for 10 s and 60 °C for 30 s, with a melt curve analysis performed from 65 °C to 95 °C to verify amplification specificity. Relative expression levels were calculated using the 2^−ΔΔCt^ method with *AtUBQ* as the internal reference.

The primers used for qRT-PCR are listed in Table S1. Among them, primers for *AtUBQ* and several target genes were adopted from previously published study [19], while primers for *AtMPK1* and *AtMPK2* were designed in this study using Oligo7 software. All new primers were validated by standard curve analysis, with only those exhibiting 90–110% efficiency being used.

### 4.4. SA Treatment

For seedling SA treatment, surface-sterilized seeds were sown on 1/2 MS medium and stratified at 4 °C in the dark for 2–3 days. They were then germinated and grown vertically at 22 °C under a 16-h light/8-h dark photoperiod for 3 days. The 3-day-old seedlings were transferred to fresh 1/2 MS medium supplemented with or without 100 μM SA and grown vertically for an additional 6 days. Phenotypic photographs were taken for analysis, and chlorophyll content was measured.

For detached-leaf SA treatment, the 6th and 7th rosette leaves of 4-week-old wild-type (Col-0) plants were excised and placed in Petri dishes containing distilled water with 0, 100, 500 μM, or 1 mM SA for 4 h at 22 °C under continuous light. After treatment, the leaves were immediately frozen in liquid nitrogen and stored at −80 °C for subsequent RNA extraction and qRT-PCR analysis.

For each treatment, at least three independent plants or seedlings per genotype were analyzed, and all experiments were performed with at least three biological replicates.

### 4.5. Measurement of Chlorophyll Content and Ion Leakage Rate

All methods for ion leakage measurement and chlorophyll content determination (using both SPAD meter and acetone extraction) were performed according to previously published protocols [19]. For chlorophyll content measurement, the 6th and 7th rosette leaves of 4-week-old wild-type plants were used. Chlorophyll content was measured using a SPAD chlorophyll meter (SPAD-502 Plus, Minolta, Tokyo, Japan), with at least three independent plants per genotype and three readings per leaf. For acetone extraction, the whole above-ground parts of 6-day-old seedlings grown on MS medium with or without SA were collected and extracted in 80% acetone at room temperature in darkness overnight. After extraction, absorbance was recorded at 645, 663, and 440 nm.

For ion leakage measurement, detached leaves from 4-week-old plants were vacuum-infiltrated in deionized water for 30 min. Initial conductivity (C1) was recorded, then the leaves were boiled for 30 min, and final conductivity (C2) was measured after cooling. Ion leakage was expressed as the ratio C1/C2.

For each assay, at least three plants or seedlings per genotype per treatment were analyzed, and all experiments were performed with at least three biological replicates.

### 4.6. Subcellular Localization

The full-length CDSs of AtMPK1 and AtMPK2 were amplified and subcloned into modified pCAMBIA1300 vectors to generate Pro35S:*AtMPK1*-EGFP and Pro35S:*AtMPK2*-EGFP constructs, respectively. Pro35S:*AtVQ25*-mCherry was used as a nuclear marker [19]. Pro35S-EGFP empty vector was used as a blank control. These constructs were transformed into Arabidopsis protoplasts. After transient expression for 12–16 h at 25 °C in the dark, fluorescence signals were detected using a laser confocal microscope (Olympus, FV3000, Tokyo, Japan). GFP fluorescence was excited at 488 nm using an argon laser, and emission was collected through a 525/50 nm band-pass filter. mCherry fluorescence was excited at 561 nm, and emission was collected through a 593/40 nm band-pass filter. Sequential scanning mode was applied to avoid cross-talk between the two channels, and all images were processed using Olympus FV31S-SW software (Ver.2.6).

### 4.7. Y2H Assay

Full-length or truncated cDNAs of AtMPK1, AtMPK2, and AtVQ25 were amplified by PCR and subcloned into the prey vector pGADT7 (AD) or the bait vector pGBKT7 (BD). The recombinant plasmids were co-transformed into yeast strain AH109. The transformed yeast cells were plated on SD/-2 medium (SD/-Trp/-Leu) to check transformation efficiency and on SD/-4 medium (SD/-Trp/-Leu/-His/-Ade) to assess protein–protein interactions. Growth on SD/-4 medium indicated positive interactions. The truncated mutants of AtVQ25 were constructed based on bioinformatic predictions of potential interaction-dependent domains (P1, P2, P3) and the conserved VQ motif.

### 4.8. LCI Assay

The LCI assay was performed following a previously described method [19]. The CDSs of AtMPK1 and AtMPK2 were cloned into the nLuc vector, and the CDS of AtVQ25 was cloned into the cLuc vector. Agrobacterium cells (GV3101) harboring different constructs were adjusted to an optical density (OD600) of 0.5–0.6 with MES buffer (10 mM MES, pH 5.7, 10 mM MgCl_2_, and 0.2 mM acetosyringone) and infiltrated into *N. benthamiana* leaves. After incubation in the dark for 48 h, D-luciferin potassium salt (LUCK-1G, GOLDBIO, St. Louis, MO, USA) was sprayed onto the infiltrated leaves. Luciferase activity was captured using a low-light cooled CCD imaging device (VILBER, FUSION FX7 Spectra, Marne-la-Vallée, France).

### 4.9. Pull-Down Assay

Recombinant proteins MBP-AtVQ25, AtMPK1-His, and GST-AtMPK2 were expressed in *E. coli* (Rosetta strain, BBI, B528439-0010, Shanghai, China) and purified using appropriate affinity resins. MBP-AtVQ25 was purified using amylose resin beads (New England Biolabs, E8021S, Ipswich, MA, USA), AtMPK1-His was purified using Ni-NTA resin (BBI, C600033-0010), and GST-AtMPK2 was purified using glutathione Sepharose beads (CYTIVA, 17075601, Marlborough, MA, USA).

For the pull-down assay between MBP-AtVQ25 and AtMPK1-His, approximately 10 μg of each purified protein was incubated in PBS buffer (50 mM Tris-HCl, pH 7.5, 50 mM NaCl, 10 mM MgCl_2_) for 30 min at 4 °C. The mixture was then incubated with amylose resin beads at 4 °C for 3 h to capture MBP-AtVQ25 and its interacting partners. After extensive washing, the bound proteins were eluted by boiling in SDS loading buffer and analyzed by Western blot using anti-MBP (TRANSGEN, HT701, Beijing, China) and anti-His (TRANSGEN, HT501) antibodies.

For the pull-down assay between MBP-AtVQ25 and GST-AtMPK2, approximately 10 μg of each purified protein was incubated in the same PBS buffer for 30 min at 4 °C. The mixture was then incubated with glutathione Sepharose beads at 4 °C for 3 h to capture GST-AtMPK2 and its interacting partners. After extensive washing, the bound proteins were eluted by boiling in SDS loading buffer and analyzed by Western blot using anti-MBP (TRANSGEN, HT701) and anti-GST (TRANSGEN, HT601) antibodies.

### 4.10. Co-IP Assay

The Co-IP assay was performed as previously described [19]. Agrobacterium strains (GV3101) harboring Pro35S:*AtVQ25*-GFP, Pro35S:*AtMPK1*-7Myc6His, or Pro35S:*AtMPK2*-3×Flag were co-infiltrated into *N. benthamiana* leaves. Total proteins were extracted from approximately 3 g of infiltrated leaf tissue homogenized in 10 mL of Co-IP buffer (50 mM Tris-HCl, pH 7.5, 150 mM NaCl, 1 mM EDTA, 0.1% Tween 20, 10% glycerol, 1 mM PMSF, and a protease inhibitor cocktail). The supernatant was incubated with 80 μL of anti-GFP magnetic beads or anti-Flag agarose beads (Smart-Life Science, SM03801, Beijing, China) for 4 h at 4 °C. After at least four washes with Co-IP buffer, the beads were boiled in SDS loading buffer. Immunoprecipitated proteins were detected by Western blot using anti-GFP (TRANSGEN, HT801), anti-Myc (TRANSGEN, HT101), or anti-Flag (TRANSGEN, HT201-01) antibodies.

### 4.11. Protein Stability and In Vitro Phosphorylation Assays

To assess the effect of AtMPK1 and AtMPK2 on AtVQ25 protein stability, total protein extracts were prepared from Pro35S:AtVQ25-7Myc6His overexpression plants. These extracts were then placed in a reaction buffer containing 25 mM Tris-HCl (pH 7.5), 10 mM NaCl, 10 mM MgCl_2_, 4 mM PMSF, 5 mM DTT, and 10 mM ATP, and incubated with either purified MBP-AtMPK1, MBP-AtMPK2, or MBP control protein, or with total protein extracts from Col-0, Pro35S:AtMPK1-GFP, or Pro35S:AtMPK2-GFP overexpression plants. For the purified protein assays, the mixtures were incubated at 25 °C for varying time intervals (0, 30, 60, 90, 120, and 150 min). At each time point, aliquots were removed, denatured in 2× SDS loading buffer, and analyzed by Western blotting using anti-Myc antibody (TRANSGEN, HT101) to monitor residual AtVQ25-7Myc6His levels. Competition assays were additionally performed by adding increasing amounts of purified GST-AtVQ25 to the reaction mixtures.

For MG132 treatment, leaves from Pro35S:AtVQ25-7Myc6His plants were frozen in liquid nitrogen and ground to a fine powder. The powder was mixed with lysis buffer and centrifuged at 12,000 rpm for 10 min. The resulting supernatant was transferred to a fresh tube and supplemented with either 2 μL of MG132 (experimental group, Sigma, SML1135, St. Louis, MO, USA) or an equal volume of DMSO (control group, Sigma, D8418). The mixtures were incubated at 25 °C, and 30-μL aliquots were collected at 0, 30, 60, 90, and 120 min. Each aliquot was mixed with 2× SDS loading buffer and analyzed by Western blotting using an anti-Myc antibody.

To verify direct phosphorylation of AtVQ25 by AtMPK1 and AtMPK2, AtMKK9 was included in the in vitro kinase reaction system to activate the kinase activities of AtMPK1 and AtMPK2. Purified GST-AtVQ25 was incubated with MBP-AtMPK1 or MBP-AtMPK2 in reaction buffer containing ATP, and phosphorylation was detected using the Phos-tag™ reagent kit (Wako, 304-93526, Osaka, Japan) following the protocol described in reference [14]. Compared with the kinase-free control, the addition of either AtMPK1 or AtMPK2 resulted in prominent mobility-shifted bands of AtVQ25, confirming that both kinases directly phosphorylate AtVQ25. For quantitative assessment of phosphorylation levels, the intensities of the shifted bands were measured using ImageJ software (Ver.2, NIH, Bethesda, MD, USA).

### 4.12. Statistical Analysis

All data are presented as mean ± SD. Statistical comparisons among more than two groups were performed using one-way analysis of variance (ANOVA), followed by Tukey’s honestly significant difference (HSD) post-hoc test for multiple comparisons. All analyses were conducted using GraphPad Prism 10.5.0. A *p*-value of <0.05 was considered statistically significant. Different letters above the bars in the figures indicate significant differences at *p* < 0.05.

## 5. Conclusions

The data establish that AtMPK1 and AtMPK2 act as upstream stabilizers of AtVQ25 through direct phosphorylation, thereby coupling MAPK signaling to SA-mediated leaf senescence. The interaction is mediated by the N-terminal and P1 regions of AtVQ25, distinct from the VQ domain used for WRKY recruitment, and is strictly required for AtVQ25 function in SA responses, as evidenced by suppression of the *AtVQ25*-OE phenotype in the *atmpk1atmpk2* background. A potential AtMPK–AtWRKY53 interaction further hints at additional regulatory complexity beyond the core AtVQ25–AtWRKY53 module. Collectively, these findings support a model in which age-dependent SA accumulation activates AtMPK1/AtMPK2, which in turn phosphorylate and stabilize AtVQ25, enabling relief of *AtWRKY53* self-repression and subsequent activation of senescence-associated genes. This study reveals a phosphorylation-dependent stabilization mechanism that integrates developmental and hormonal signals into the VQ-WRKY transcriptional hub, providing a framework for understanding the onset of leaf senescence.

## Data Availability

Data are contained within the article and Supplementary Material.

## Supplementary Materials

The following supporting information can be downloaded at https://www.mdpi.com/article/10.3390/plants15172726/s1, Figure S1: Subcellular Localization of AtMPK1 and AtMPK2; Figure S2: STRING Database Prediction of Interactions between AtMKK9 and AtMPK1/AtMPK2; Figure S3: Phenotype of Col-0, *atmpk1*, *atmpk2*, and *atmpk1atmpk2* seedlings under SA treatment. Figure S4: Y2H Assay of AtWRKY53 with AtMPK1/AtMPK2 and Prediction of Phosphorylation Sites; Table S1: Primers used in this study.
