# Supplementary material for: AtMPK1/AtMPK2 Interact with AtVQ25 to Enhance Its Protein Stability and Coordinately Regulate Salicylic Acid-Mediated Leaf Senescence
Source: Plants (Basel). 2026 Sep 6;15(17):2726. doi: 10.3390/plants15172726 (PMC13568464; doi:10.3390/plants15172726)
Supplement: Supplementary file 1 [file plants-15-02726-s001.zip › plants-4486911-supplementary.pdf]

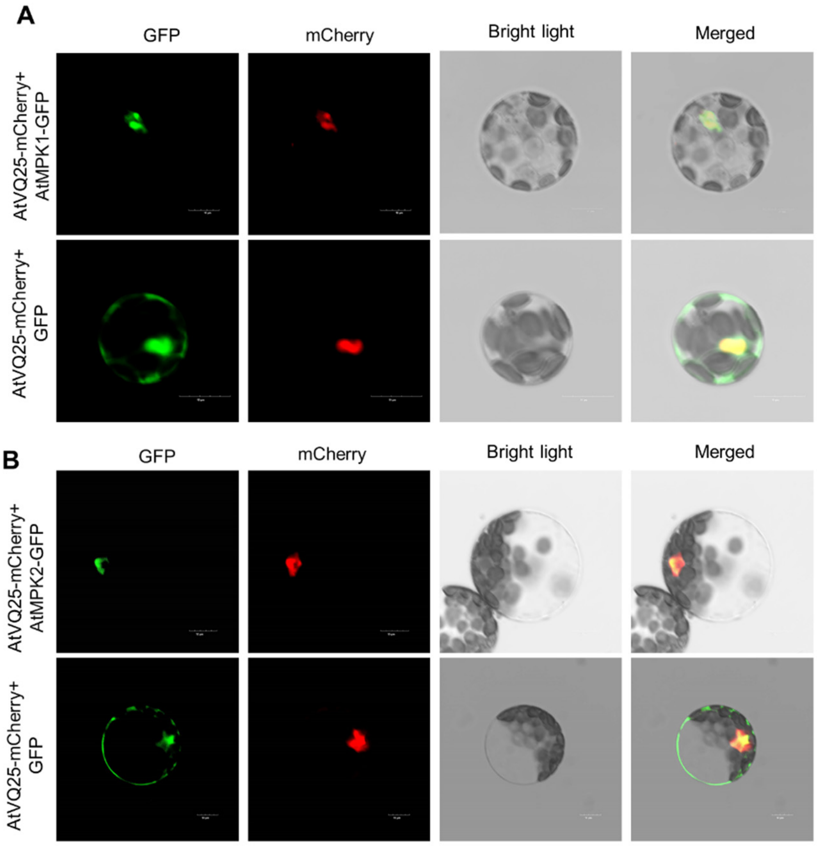

Figure S1. Subcellular Localization of AtMPK1 and AtMPK2.

(A, B) Confocal laser scanning microscopy images of Arabidopsis protoplasts transiently expressing AtVQ25-mCherry, AtMPK1-EGFP, AtMPK2-EGFP, or EGFP alone. Bars = 10 μm. All experiments were repeated at least three times with similar results.

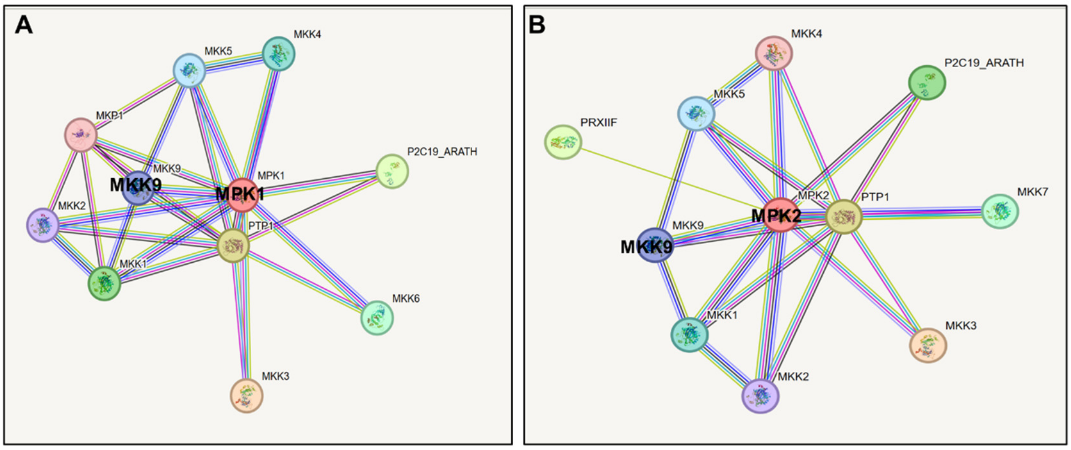

Figure S2. STRING Database Prediction of Interactions between AtMKK9 and AtMPK1/AtMPK2.

(A) Predicted interaction network between AtMKK9 and AtMPK1 from the STRING protein–protein interaction database. Line thickness indicates the predicted confidence score of the interaction. **(B)** Predicted interaction network between AtMKK9 and AtMPK2 from the STRING database. Line thickness indicates the predicted confidence score of the interaction.

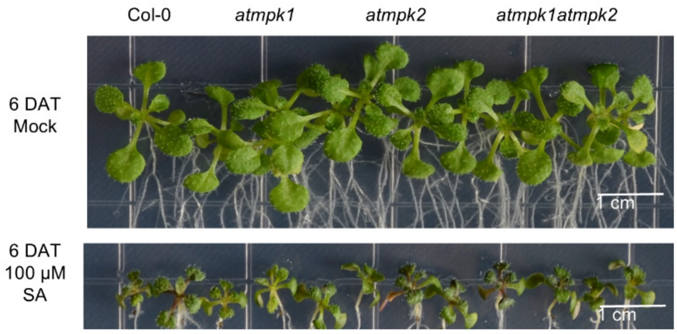

Figure S3. Phenotype of Col-0, *atmpk1*, *atmpk2*, and *atmpk1atmpk2* Seedlings under SA treatment.

Phenotypes of Col-0, *atmpk1*, *atmpk2*, and *atmpk1atmpk2* seedlings grown on MS medium with or without 100 μM SA for 6 d. Scale bar = 1 cm. All experiments were repeated at least three times with similar results.

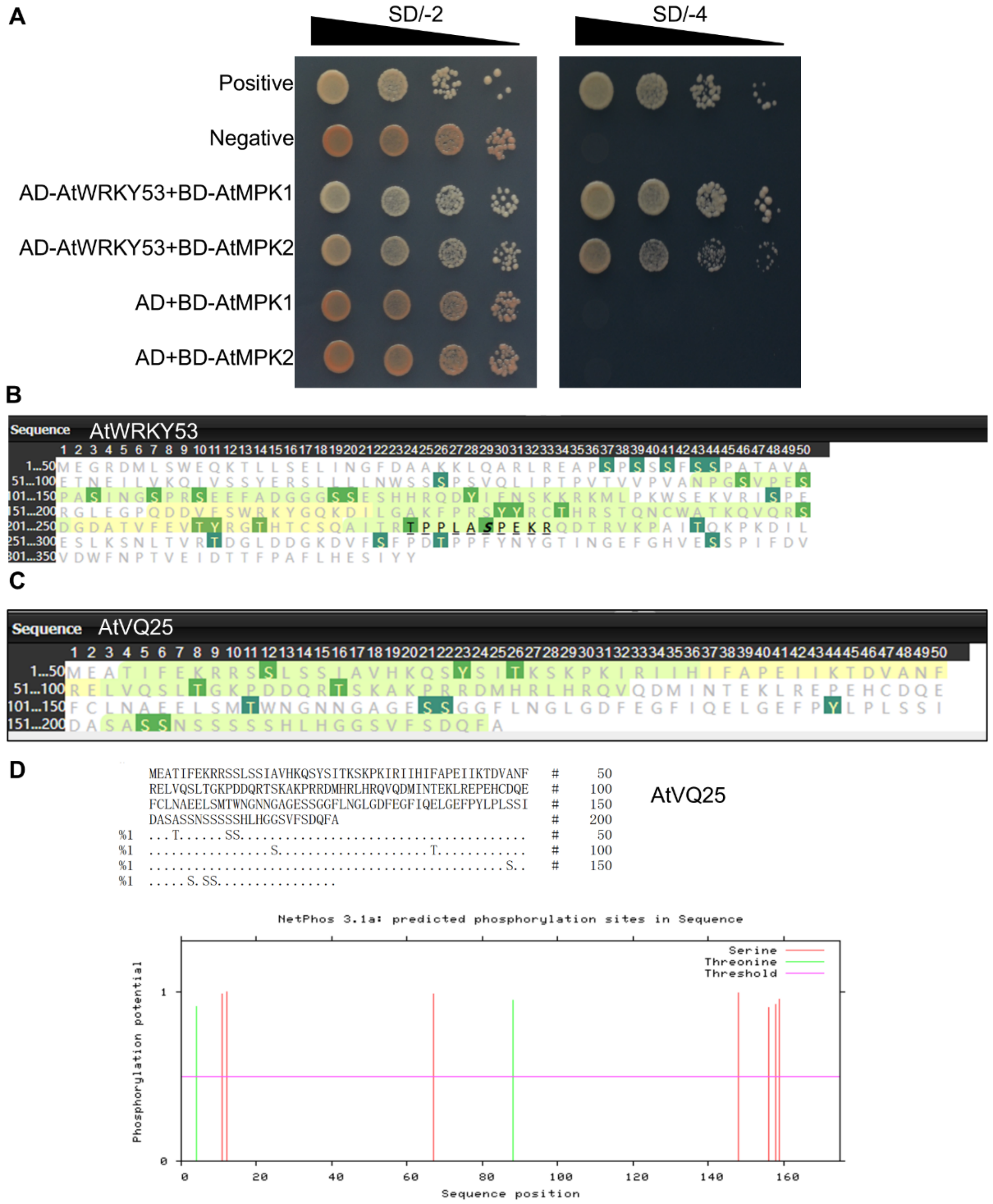

Figure S4. Y2H Assay of AtWRKY53 with AtMPK1/AtMPK2 and Prediction of Phosphorylation Sites.

(A) Y2H assay detecting the interaction of AtWRKY53 with AtMPK1 and AtMPK2. Transformants were grown on SD/–Trp/–Leu and on SD/–Trp/–Leu/–His/–Ade. AD-T/BD-p53 and AD-T/BD-Lam were used as positive and negative controls, respectively. All experiments were repeated at least three times with similar results.

(B) Prediction of potential phosphorylation sites in the full-length AtWRKY53 protein by the PhosPhAt database. **(C)** Prediction of potential phosphorylation sites in the full-length AtVQ25 protein by the PhosPhAt database. **(D)** Prediction of potential phosphorylation sites in the full-length AtVQ25 protein by the NetPhos-3.1 server.

Table S1. Primers used in this study.

Primers used in this study		
AtMPK1-QRT-F	CTAAGTACATGCCTATCAAGCC	For qRT-PCR of AtMPK1
AtMPK1-QRT-R	TTACCAGGTTTCAAATCTCGG	For qRT-PCR of AtMPK1
AtMPK2-QRT-F	AATACGTGCCTATCAAACCGA	For qRT-PCR of AtMPK2
AtMPK2-QRT-R	GGTTACCGGGTTTCAGATCC	For qRT-PCR of AtMPK2
AtMPK1-F	CTCTAGAATGGCGACTTTGGTTGATCCTC	For cloning CDS of AtMPK1
AtMPK1-R	GACGCGTATGGCGACTTTGGTTGATCCTC	For cloning CDS of AtMPK1
AtMPK2-F	GAATTCATGGCGACTCCTGTTGATCCA	For cloning CDS of AtMPK2
AtMPK2-R	GTCGACAAACTCAGAGACCTCATTGTTGT	For cloning CDS of AtMPK2
AtMKK9-F	GGAATTCATGGCTTTAGTACGTGAAC	For cloning CDS of AtMKK9
AtMKK9-R	GCGTCGACAAGATCTTCCCGGAGAAAAGGAT	For cloning CDS of AtMKK9
atmpk1-LP	AATGGGATAAGGAATGAAGGG	For identify atmpk1 T-DNA mutant
atmpk1-RP	TGCATTCGGGTCATATAGAGG	For identify atmpk1 T-DNA mutant
atmpk2-LP	TGCACTCGGGTCATATAAAGG	For identify atmpk2 T-DNA mutant
atmpk2-RP	ATGTGGCAAACACTTTTCGAG	For identify atmpk2 T-DNA mutant
LBb1.3	ATTTTGCCGATTTCGGAAC	For identify SALK T-DNA mutant
AtUBQ-QRT-F	CAAATCCAAAACCCTAGAAACCGAA	For qRT-PCR of AtUBQ30
AtUBQ-QRT-R	ATCTCCCGTAGGACCTGCACTG	For qRT-PCR of AtUBQ30
